# Editorial: Adaptive networks in functional modeling of physiological systems

**DOI:** 10.3389/fnetp.2022.996784

**Published:** 2022-08-25

**Authors:** Eckehard Schöll, Jakub Sawicki, Rico Berner, Plamen Ch. Ivanov

**Affiliations:** ^1^ Institut für Theoretische Physik, Technische Universität Berlin, Berlin, Germany; ^2^ Potsdam Institute for Climate Impact Research, Potsdam, Germany; ^3^ Bernstein Center for Computational Neuroscience Berlin, Humboldt-Universität, Berlin, Germany; ^4^ Fachhochschule Nordwestschweiz FHNW, Basel, Switzerland; ^5^ Institut für Physik, Humboldt-Universität zu Berlin, Berlin, Germany; ^6^ Keck Laboratory for Network Physiology, Department of Physics, Boston University, Boston, MA, United States; ^7^ Harvard Medical School and Division of Sleep Medicine, Brigham and Women’s Hospital, Boston, MA, United States

**Keywords:** adaptive networks, functional modeling, network physiology, dynamical systems, cluster synchronization, coupled oscillators, pattern formation

Adaptive networks provide a paradigm to investigate natural phenomena in fields ranging from physics, chemistry, biology and neuroscience to physiology, medicine and socio-economic systems (Ivanov, 2021). A fundamental problem is to understand how global behaviors emerge out of interactions among dynamically changing entities with coupling forms that are nonlinear and vary as functions of time. This is particularly relevant in the field of network physiology (Bashan et al., 2012), where links in dynamic networks of physiological interactions represent coordination and synchronization between systems and sub-systems, and exhibit transient characteristics (Bartsch et al., 2015). A key question is how states and functions emerge out of the collective network dynamics of integrated systems. While network structure may play a role in generating various states and functions, different global behaviors can emerge from the same network topology due to changes in systems dynamics and modulations in the functional form of interactions. Changes in the output dynamics of a system can propagate via “elastic” time-varying links to affect the dynamics of other systems, and thus, alter the behavior of the entire network. Further, coupling and synchronization patterns modulate with transitions across states in response to changes in physiologic regulation (Bartsch et al., 2012; Liu et al., 2015), while systems can communicate through several forms of coupling that simultaneously coexist (Bartsch et al., 2014a; Bartsch et al., 2014b). This poses new challenges in developing generalized methodology adequate to quantify such systems and their network interactions. Recent advances in adaptive networks, where connectivity changes in time, offer a promising framework to explore how network structure reorganizes adaptively in response to systems dynamics (Gross and Blasius, 2008; Berner, 2021). One of the simplest forms of adaptation can be found in the synchronization of coupled nonlinear oscillators. Various synchronization patterns are known, like cluster synchronization where the network splits into groups of synchronous elements, or partial synchronization patterns like chimera states where the system splits into coexisting domains of coherent (synchronized) and incoherent (desynchronized) states. These patterns are also explored in adaptive networks, where several dynamical scenarios have been revealed including the self-organized formation of co-existing frequency clusters or chimera-like states. This Research Topic features contributions within a general dynamical systems perspective, and a view to applications in physiological systems, in order to shed light on the complex interplay between adaptivity induced phenomena with complex connectivity structures, coupling delay, and noise. The focus is on functional modelling of the interactions between different systems in the living organism, not on a detailed biochemical modelling of a single organ or system. The Research Topic includes adaptive networks in neuroscience, adaptive interactions, communication and information exchange between cells and organs, partial synchronization patterns in single and multilayer networks, and control and regulatory mechanisms in adaptive networks. The goal of this Research Topic is to present recent progress in the field of networks of dynamical systems, outline future challenges and provide an overview of the state of the art. Since adaptive networks have only recently become a major focus of research, some of the contributions may open routes to extend the present work by considering an adaptive network structure as an intriguing future research perspective. We hope that this Research Topic will inspire, inform and provide direction and guidance to researchers in the field.

The first two articles in the Research Topic consider general synchronization phenomena, while the other four focus on applications in microbial networks, in neuronal systems, and in human physiological networks with the immune system as a reference point.

Saha and Dana investigate the dynamics in a system of three identical oscillators coupled in a ring with repulsive interactions. The individual dynamics of each oscillator is described by superconducting Josephson junctions where each node’s state is expressed by phase and voltage variables. Following recent research on adaptive networks (Berner et al., 2021), this system of Josephson junctions could be interpreted as an adaptive system for the phase variables. In this work, various dynamical patterns including chimera states are observed. The chimera pattern first appears in chaotic rotational motion of the three junctions when two junctions evolve coherently, while the third junction is incoherent. For larger repulsive coupling, the junctions evolve into another chimera pattern in a periodic state when two junctions remain coherent in rotational motion and one junction transits to incoherent librational motion. A map of regimes for the system’s damping parameter and the repulsive coupling constant shows the parameter regions for which the different states exist. This map is further augmented by the stability boundaries of the synchronous states from a master stability approach. With the numerical and analytical results, this work provides insights on the impact of repulsive coupling for the emergence of partial synchronization patterns in coupled systems of Josephson junctions.

Franović et al. study the collective bursting dynamics in a population of excitable units adaptively interacting with a pool of resources. With this they propose a simple paradigmatic model to study the emergence of complex collective phenomena induced by a dynamically co-evolving pool of (metabolic) resources. In this work, the resource pool is considered to be influenced by the average activity of the population, whereas the feedback from the resources to the population is comprised of components acting homogeneously or inhomogeneously on individual units of the population. Moreover, the resource pool dynamics is assumed to be slow and has an oscillatory degree of freedom. The authors show how this complex interplay between the population and the resources gives rise to collective activity bursting which is characterized by a recurrent switching between episodes of quiescence and episodes of activity bursts in the population of active rotators. To obtain an analytical understanding for collective activity bursting, they use the Ott-Antonsen reduction for the collective dynamics of the population and singular perturbation theory for the slow flow of the resources. Further, it is shown that the collective activity bursting may stably coexist with a steady state. As one of these regimes could be desirable or undesirable from the applied point of view, Franović et al. discuss two simple approaches that can successfully induce switching between the two regimes.

Lücken et al. investigate how complex cross-feeding networks can serve as a basis for exploring natural evolutionary and radiation processes in microbial ecosystems. A characteristic attribute of many ecological networks in the microbial domain is the diversity of substrates, which is an important prerequisite for the immense biodiversity in microbial ecosystems. Using an in-depth numerical analysis, the authors are able to show how microbial species can evolve from a simple initial community, taking into account some elementary evolutionary mechanisms of resource-dependent speciation and extinction. Even in stable environments, the system is subject to persistent turnover, suggesting ongoing co-evolution. In this context, the diversity of the system is self-sustaining because new microbes produce new substrates, which in turn allow new microbes to colonize. A central concern of the authors is to uncover the influence of various parameters, such as mineralization ratio, and metabolic versatility and variability, on the emergent structural properties and substrate formation. This transformation and accumulation of substrates is also found in natural systems such as soils and oceans, which may be important for further biotechnological applications.

Khaledi-Nasab et al. investigate the long-lasting effects of coordinated reset stimulation in networks with leaky integrate-and-fire neurons with spike-timing-dependent plasticity. Coordinated reset stimulation is a theory-based stimulation technique designed to counteract neuronal synchrony through desynchronization. Synchronized neuronal activity is commonly associated with various neurological disorders, including essential tremor and Parkinson’s disease. Coordinated reset is a procedure in which phase-shifted stimuli are delivered to multiple neuronal subpopulations for the treatment of refractory Parkinson’s disease. Computational studies of coordinated reset stimulation of plastic neuronal networks demonstrate long-lasting desynchronization effects achieved by down-regulating abnormal synaptic connectivity. In this way, networks are moved into attractors with more stable desynchronized states, so that stimulation-induced desynchronization persists even after stimulation is terminated. A simple model with 103 excitatory leaky integrate-and-fire neurons with spike-timing-dependent plasticity is used for the study. The low computational cost allows the authors to explore the parameter space in detail. Together with analytical calculations, the study shows long-lasting desynchronization effects of coordinated reset stimulation with and without randomization of stimulus amplitudes alone, with randomization of stimulus times alone, and with a combination of both. By varying the coordinated reset stimulation frequency relative to the frequency of the abnormal target rhythm and the number of neuronal subpopulations stimulated separately, the study reveals parameter regions and associated mechanisms in which the two qualitatively different randomization mechanisms enhance the robustness of the long-lasting desynchronization effects of coordinated reset. The results have potential relevance for long-lasting therapeutic effects of randomized brain stimulation.

Anesiadis and Provata investigate synchronization phenomena in a multiplex network consisting of two layers. Each layer is represented by a ring of non-locally coupled identical leaky integrate-and-fire neurons. On the other hand, there are one-to-one interactions between the layers. This model represents the two hemispheres of the brain, where within each hemisphere the different brain regions interact only with neighboring regions, while between hemispheres each region is primarily connected to the functionally homologous region. The authors consider both excitatory and inhibitory connections. Different parameter regimes are examined in the numerical study, using the Kuramoto order parameter as an index of synchrony within each ring and the correlation function as an indicator of synchrony between rings. The study shows that the dynamics of the multiplex network can range from coexistence of active and subthreshold domains, solitary states, chimera states, to complete coherence or incoherence. In particular, when there exists weak coupling between the two layers (weak multiplexing), different synchronization patterns are supported in the system of the two rings. These are stable and are found when the values of the intra-ring coupling are close to the critical points separating qualitatively different synchronization regimes, e.g., between the regime of traveling fronts and that of chimera states.

Sawicki et al. provide a dynamical systems perspective to the modelling of pathological states induced by tumors or infection. A unified disease model is established using the innate immune system as the reference point. The authors propose a two-layer network model for carcinogenesis and sepsis based upon the interaction of parenchymal cells and immune cells via cytokines, and the co-evolutionary (adaptive) dynamics of parenchymal, immune cells, and cytokines. The interaction of parenchymal cells (metabolism) and the nonspecific immune cells (reaction of innate immune system) are represented by nodes of a duplex layer. The cytokine interaction is modeled by adaptive coupling weights between the nodes. Using this functional model, the authors explain carcinogenesis, tumor progression, and sepsis by destabilization of the healthy homeostatic state (frequency synchronized), and emergence of a pathological state (desynchronized or multifrequency cluster). Thereby, carcinogenesis, organ dysfunction in sepsis, and recurrence risk can be described in a correct functional context. As a guiding example for future work, the authors analyze and interpret the emerging dynamical phenomena due to the presence of pathological cells (tumor) or a dysregulated immune system (sepsis) in dependence on a physiological sum parameter called the age parameter. In summary, the study of Sawicki et al. opens a perspective for further dynamic disease modeling in the context of network physiology.
